# Text encryption through bio-inspired DNA and RNA sequencing

**DOI:** 10.1371/journal.pone.0345090

**Published:** 2026-04-08

**Authors:** Omar Fitian Rashid, Safa Ahmed Abdulsahib, Humam Al-Shahwani

**Affiliations:** 1 Department of Geology, College of Science, University of Baghdad, Baghdad, Iraq; 2 Department of Computer Science, College of Science, University of Baghdad, Baghdad, Iraq; University of Electronic Science and Technology of China, CHINA

## Abstract

Cryptography is the technology of protecting information and communication by means of encoding the sending information. Existing methods often operate within predetermined mathematical structures which can be subject to pattern recognition and which are lack biological randomness. To address these challenges, this paper introduced a new bio-inspired cryptography approach to protect text messages using the coding mechanisms of DNA and RNA materials. The encryption system consists of six steps: encoding the plaintext message into DNA sequences, transcribing DNA into RNA, applying the complementary base pairing, scrambling RNA segments, optional reverse transcription, and encoding the resulting DNA-RNA characters into ciphertext. Randomization is used in the encoding of DNA and in the scrambling of RNA to make each encryption instance different from the other. This method was tested with varying sizes of messages, and it has proved very efficient in terms of time between encryption and decryption. This approach has created new opportunities for the creation of new classes of secure cryptographic systems bio-inspired to get modern methods of digital communication.

## 1. Introduction

**Fig 1:**
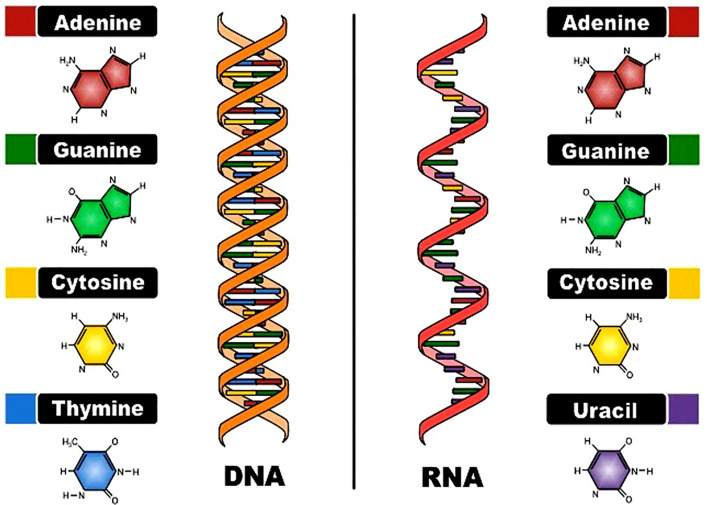
The structure of DNA and RNA.

DNA is a main molecule which codes all known forms of life and many viruses. Its structure, a double helix composed of nucleotides, allows it to encode biological information in sequences of four bases: these are Adenine (A), Thymine (T), Cytosine (C) and Guanine (G). This code defines how organisms evolve, how they operate and how they are reproduced from one generation to the other by providing the details of how such organisms can be constructed [1]. While RNA, or ribonucleic acid, is an important molecule in biology which has its own identity. Whereas DNA holds the genetic information, RNA actively translates those instructions to proteins which are essential for cellular functions. One significant feature of RNA is that it is single-stranded and has a base called uracil (U), not the present in DNA called thymine (T). The functionality of RNA is owing to the fact that it can carry the genetic code from the nucleus to the cytoplasm and then convert this code into functional proteins [2]. The structure of DNA and RNA is illustrated in Fig 1 [3].

On the other hand, cryptography is the practice and theory of protecting information by putting it in a form that cannot be understood without proper permission [4]. Classical methods of secure communications are based on the use of mathematical calculations to encode and decode information and to provide protection against interception and alteration, as well as to check the sender’s identity. Cryptography has turned into an essential component of cybersecurity as the use of digital technologies grows rapidly and makes it possible to secure information from unauthorized access [5]. Since biological sciences are increasingly being combined with computer science, especially in the field of digital security, researchers have begun to consider how DNA and RNA can be incorporated into cryptographic systems as the basis for data protection. DNA cryptography, for instance, the properties of DNA sequences can be used to encode the message into a secure data format. Specifically, DNA cryptography is an attempt to use the features of DNA as an information storage medium to establish a method of data protection that is different from encryption. In the same way, RNA-based systems are considered to be used for dynamic and responsive encryption techniques, as RNA molecules can easily transform themselves, which will create extra layers of protection. The use of DNA and RNA in cryptography is helpful because, without molecular biological tools, it is very difficult to decipher the biological sequences [6–7]. However, even now, DNA and RNA cryptography are considered rather new, and some issues have to be solved. Some of the challenges that are currently hindering their use include high costs, technical difficulties, and the requirements of specialized apparatus. While these forms of cryptography are still complex today, as accessible biotechnology grows, the scientific community discovers better methods of synthesizing DNA and RNA, making it probable that they will bring these forms of cryptography to the forefront. However, the combination of biological sciences and cryptography is one of the most promising directions in the field of data protection, as well as providing new approaches to modern information technologies.

In various publications research, DNA cryptography methods are proposed and are employed in protecting data to be transferred over the internet. Almasoud et al. [8] proposed a new image encryption method to enhance security based on improved Bonobo Optimizer and DNA coding, where the proposed method consists of initial value generation, substitution, diffusion, and decryption. DNA encoding is utilized to get encrypted images. A hybrid cryptosystem method is proposed by [9] by using the concepts of DNA cryptography, where the proposed method generates a random key with various DNA encoding and uses a mealy machine to enhance the security. A new bio-inspired cryptosystems method to secure data is proposed [10], where this method is done by converting the binary to DNA sequences, then converting DNA to mRNA and finally converting mRNA to Protein. Kumar [11] proposed a secure architecture to secure data on cloud servers by using DNA cryptography, HMAC, and a third-party Auditor, where various cryptographic algorithms are investigated. A novel image encrypting method is suggested by [12]; this method is based on the developed Vigenere algorithm by merging the tent map with the logistic map. A new chaotic image encryption method is presented by using DNA coding and RNA computing [13], where this method starts by creating a four-dimensional hyperchaotic model, encoding the plaintext image, and then applying RNA coding conversion and amino acid substitution box generation. A new method is proposed to get a secure information security platform by using DNA cryptography and the AES method where these methods represent a technologically great option [14]. Karthikeyan and Poongodi [15] proposed a new secure data transmission method in smart cities, where this method used an LZW lossless compression algorithm to compress the data and then encrypt it using DNA cryptography. Erkan et al. [16] proposed a new image encryption based on three-dimensional Xin-She Yang map, where this model consist of 3D hyperchaotic system-driven Multi-Layer Multi-Directional IE algorithm. A new chaos-driven image encryption method is suggested by [17] utilizing a four-dimensional Henon memristor map. A multi-layered security model is proposed by integrating cryptography and multi-image steganography in order to strengthen text protection during transmission [18]. The most recent developments in the area of DNA and RNA cryptography have examined the nonlinear dynamics, quantum chaos, and chain feedback of securing image and face data [19–22]. In contrast to these strategies that work mainly with the multimedia data, the suggested strategy aims at secure text messaging based on the multi-stage biological transformations and on the randomness depending on the session. This difference makes the proposed scheme a lean but a very versatile alternative of the wider DNA cryptography context.

Several recent studies have shown weaknesses of chaos-DNA-based encryption schemes to both chosen-plaintext and differential attacks [23–24]. The suggested cryptosystem is resistant to these weaknesses as it uses session-specific DNA coding tables and RNA segment scrambling, which removes fixed codes. Also, the optional reverse-transcription step adds hybrid biological representations, which increases resistance to known cryptanalysis methods against static DNA operations. The most recent internet progress in secure data communication has been more biased towards the lightweight cryptographic systems, chaos-based encryption and adaptive security systems that are optimized to execute in resource-constrained and distributed settings. Specifically, recent works in the fields of IoT security and data-centric protection have shown that a combination of dynamic encoding schemes and the use of nonlinear transformation layers with complexities can significantly boost resistance to cryptanalysis and still be computationally efficient [25–27]. Moreover, the security architectures that use adaptive and context-aware encryption have demonstrated better resistance to changes in attack model in large scale communication systems [28–29]. Encouraged by these advancements, DNA and RNA-based bio-inspired cryptography has become one of the most promising alternatives providing high randomness, large key space, and structural and statistical attack resistance attributes through biologically-inspired encoding and transformation models.

## 2. Materials and methods

**Fig 2:**
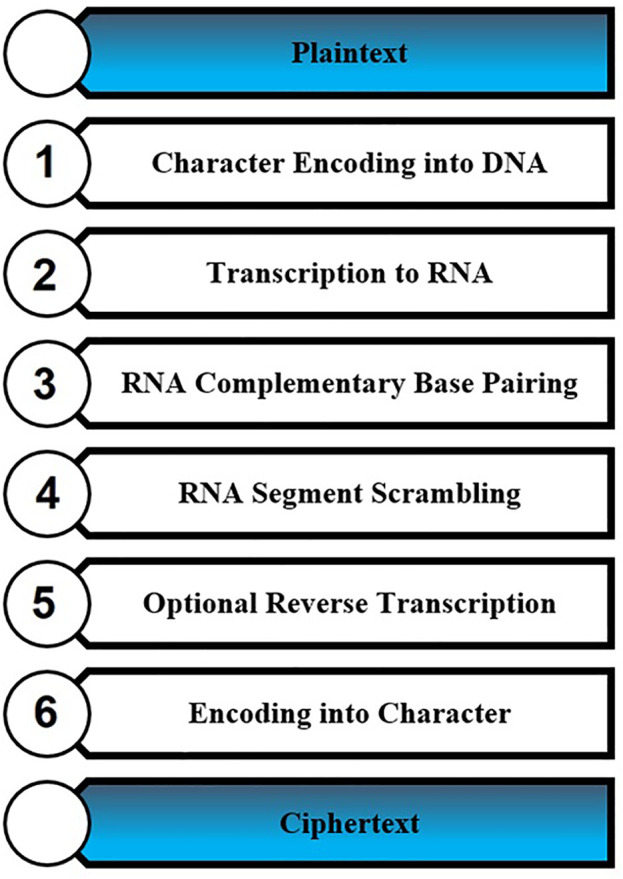
Text encryption steps.

The presented text cryptography method is utilized in this work to secure message transmission within the internet. The presented cryptography method is based on six steps and these steps are illustrated in Fig 2.

On the sender side, the encryption of sending a message is done within six steps, where the first step of the proposed method is plaintext (message) encoding into DNA sequences by building a DNA encoding table to represent all possible message characters and using four random DNA characters to represent these possible characters, where four DNA characters can represent 128 different characters. Encoding is generated randomly every time a new message is sent. The build character encoding into DNA is listed in Table 1.

**Table 1:** Character encoding into DNA.

Message	DNA encoding	Message	DNA encoding
a	GGGC	M	ATGT
b	CTAT	N	TCGA
c	TGAC	O	GCTA
d	TTCT	P	TGCA
e	TATC	Q	CATA
F	GTAG	R	ACGT
g	CCGG	S	GCTT
h	CCTT	T	GGTC
i	GTTA	U	CCGA
j	ATGA	V	CGTG
k	GAAT	W	CGAG
l	CACC	X	CGTC
m	CTTC	Y	AGCT
n	CAAG	Z	CAGG
o	CCGC	1	GTAT
p	GAAG	2	ATAA
q	ACTC	3	TCCA
r	TCCT	4	AAAG
s	GGCA	5	GGAC
t	GGTT	6	GTCT
u	AGTC	7	GAGG
v	CCCA	8	ACCC
w	ATAC	9	TTGC
x	CCAC	Space	GCCC
y	CGGA	(	TAGC
Z	TGGC	)	AAAC
A	ATCT	@	TGCT
B	GTAA	&	GAGT
C	AACA	$	GTGC
D	AGGG	%	GATG
E	TAAG	?	TAGT
F	CCAG	!	TCTT
G	TGAT	+	GTTC
H	CAGA	–	ATTG
I	TACC	/	CAAA
J	TTCG	*	GCAC
K	CAAC	,	ACAT
L	TCAC	.	GCCT

The second step is transcription to RNA, where the achieved DNA sequences from the previous step are converted to RNA characters; where the main difference between encoding using DNA and RNA, the DNA consists of adenine (A), cytosine (C), guanine (G), and thymine (T). In contrast, RNA consists of adenine (A), cytosine (C), guanine (G), and uracil (U).

The third step is RNA complementary base pairing; this is done by converting each RNA character to its complementary, as shown in Table 2.

**Table 2:** RNA characters and their complementary.

RNA character	Complementary
A	U
C	G
G	C
U	A

The fourth step of the encoding method is RNA segment scrambling, where this step starts by dividing the RNA into segments such as (AACA, CGUG, UCCG, and GACA) and then rearranging these segments based on a random key. For example, the achieved sequences after scrambled are “UCCG AACA GACA CGUG.”

The fifth step is optional reverse transcription, where a part from RNA sequences achieved from the previous step is encoded to DNA sequences, where the odd position is changed to DNA, while the even position is not changed and kept as RNA, i.e., if any odd position includes “U” will be changed to “T”. An optional reverse engineering security mechanism is added as reverse transcription. It enables deterrent cryptanalysis by heterogenizing ciphertext by mixing DNA and RNA representations to make cryptanalysis more difficult. This step is suggested in case of applications that emphasize on maximum security. On the other hand, in applications that are very time sensitive and demand minimum overhead the step can be omitted without interfering with the accuracy of the encryption and decryption process. This flexibility enables the proposed system to enforce security and efficiency depending on the needs of the application.

The last step is encoding the DNA-RNA characters obtained from the fifth step into alphabet characters, and this step starts by building a random encoding table to encode each two DNA-RNA characters into a random alphabet character, where the built encoding table is illustrated in Table 3.

**Table 3:** DNA-RNA characters encoding.

DNA-RNA	Character	DNA-RNA	Character
AA	E, m	GT	A, s
AC	P, i	GU	N, k
AG	D, Z	TA	K, p
AT	T, e	TC	B, d
AU	J, q	TG	Y, t
CA	C, n	TT	R, l
CC	S, f	TU	W, x
CG	F, a	UA	O, r, y
CT	M, u	UC	H. g
CU	X, j	UG	L, w
GA	Q, c, z	UT	V, b
GC	I, v	UU	G, o
GG	U, h		

The text that is achieved from the last step will represent the final encryption message (ciphertext) that will be sent to the receiver. In order to be highly random and reproducible, the proposed encryption framework uses two independent secret keys. The former randomizes the DNA encoding table, and each plaintext character is encrypted by an identical random choice among the set of four base sequences constructed by the use of a uniform random selection mechanism over. The mapping is recreated every time an encryption session occurs so that the same plaintext messages produce different encrypted messages in different sessions. The second phase, where the governance of the RNA segment scrambling phase. Complementary RNA transformation is followed by division of the RNA sequence into fixed-length segments, the permutation order of which is computed using a key-dependent scrambling rule. This key is safely shared between the receiver and the sender and both need this key to properly de-assemble original segment order to decrypt it. Encoding randomness together with segment scrambling contributes to high-resistance against pattern analysis and replay attacks to a great extent. The proposed encryption steps are illustrated in Algorithm 1.

**Algorithm 1. Proposed text encryption steps**

**Input:** Plaintext (Message)

**Output:** Ciphertext

Insert the message and encryption key.

Msg = read ()

Key = read ()

Build DNA sequences table.

For i = 1 To all possible values

For j = 1–4

C = Int (Rand * 4)

If C = 0 then DNA (i) = DNA (i) & “A”

If C = 1 then DNA (i) = DNA (i) & “C”

If C = 2 then DNA (i) = DNA (i) & “G”

If C = 3 then DNA (i) = DNA (i) & “T”

End for

End for

**Step 1: Character Encoding into DNA**

For L = 1 To Msg_Length

For j = 1–4

x = Substring (DNA (j), L, 1)

For i = 1 To all possible values

If x = Character (i) then DNA_Seq (L) = DNA (i)

End for

End for

**Step 2: Transcription to RNA**

For i = 1 To Msg_Length

For j = 1–4

x = Substring (DNA(i), j, 1)

If x = “A” Then

RNA(i) = RNA(i) & “A”

End If

If x = “C” Then

RNA(i) = RNA(i) & “C”

End If

If x = “G” Then

RNA(i) = RNA(i) & “G”

End If

If x = “T” Then

RNA(i) = RNA(i) & “U”

End If

Next

**Step 3: RNA Complementary Base Pairing**

For i = 1 To Msg_Length

For j = 1–4

x = Substring (RNA(i), j, 1)

If x = “A” Then

CRNA(i) = CRNA(i) & “U”

End If

If x = “C” Then

CRNA(i) = CRNA(i) & “G”

End If

If x = “G” Then

CRNA(i) = CRNA(i) & “C”

End If

If x = “U” Then

CRNA(i) = CRNA(i) & “A”

End If

Next

**Step 4: RNA Segment Scrambling**

For i = 1 To Msg_Length Step Key

c = 0

For j = Key To 2 Step −1

SRNA(i + c) = CRNA(i + j – 1)

c = c + 1

Next

SRNA(i + c) = CRNA(i)

Next

**Step 5: Optional Reverse Transcription**

For i = 1 To Msg_length

x = Substring (SRNA(i), i, 1)

If i Mod 2 <> 0 Then

s = x

If x = “U” Then

s = “T”

End If

ORT = ORT & s

End If

x = Substring (SRNA(i), i, 1)

If i Mod 2 = 0 Then

s = x

ORT = ORT & s

End If

Next

**Step 6: Encoding into Character**

For i = 1 To ORT_length step 2

x = Substring (ORT, i, 2)

For j = 1 To encoding table values

If x = DNA_RNA (j) then Cip (i) = chr (j)

End for

End for

**Fig 3:**
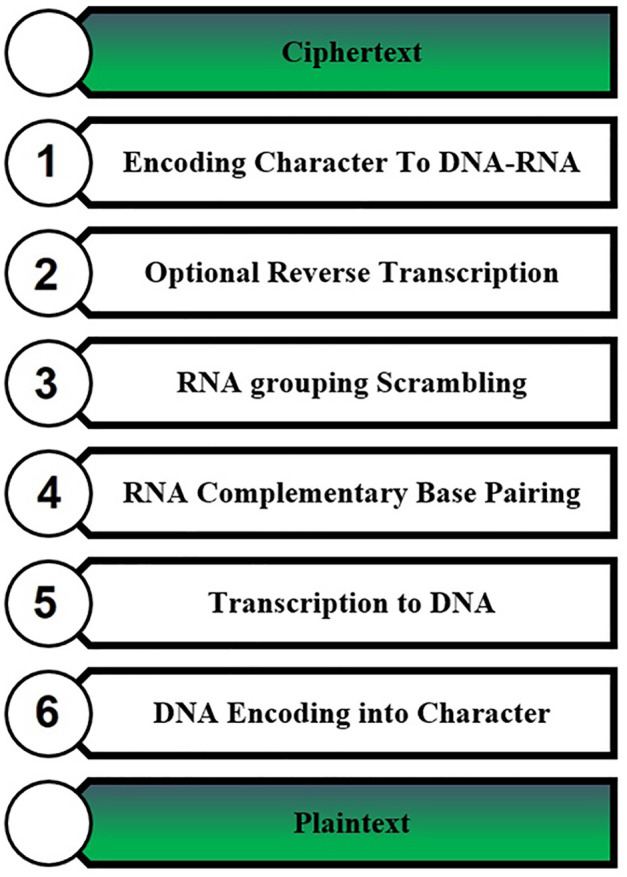
Text decryption steps.

On the receiver side, after receiving the message (ciphertext) a decryption process is applied on the message, where the decryption process is shown in Fig 3.

Where the decryption process is achieved by:

- Receive the message from the sender.
- Convert alphabet characters to DNA-RNA characters using Table 3.
- Reverse transcription of odd positions and convert DNA characters to RNA characters.
- RNA grouping scrambling by rearranging and grouping the RNA blocks.
- Convert each RNA character to its complementary.
- Convert RNA characters to DNA characters.
- Finally, the DNA sequences convert to alphabet characters, and the obtained characters are the original message.

## 3. Results and discussion

**Fig 4:**
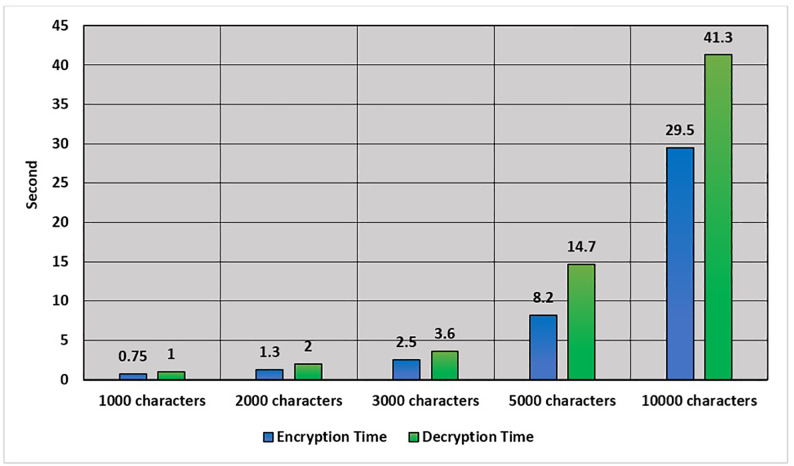
Cryptography time in terms of seconds.

In order to increase the reliability of the results, ten repetitions of encryption and decryption experiments were done under each message size. The reported time values are the average execution time with the standard deviation. The findings show that the variance of the proposed algorithm is low among the trials, which proves the consistency of the algorithm. Recent privacy preserving encryption algorithms using nonlinear dynamics and transform-domain processing have used similar statistical evaluation strategies, which proves the validity of the experimental methodology. In the proposed cryptography method, the cryptography process time is important; therefore, the achieved cryptography process time in terms of seconds using different message sizes is declared in Table 4 and Fig 4.

**Table 4:** The mean and standard deviation of the encryption and decryption time when run 10 times using various message sizes.

Message length	Encryption Time (s)	Decryption Time (s)
1000 characters	0.75 ± 0.04	1 ± 0.06
2000 characters	1.3 ± 0.07	2 ± 0.09
3000 characters	2.5 ± 0.11	3.6 ± 0.15
5000 characters	8.2 ± 0.26	14.7 ± 0.41
10000 characters	29.5 ± 0.88	41.3 ± 1.12

Table 4 presents the results of the standard deviation where all standard deviation values are relatively small in relation to the respective mean execution times, which means that the variance of repeated trials is low. This proves the stability and deterministic performance of the proposed algorithm even though randomization is used in DNA coding and RNA scrambling. The approach of similar statistical assessment has been followed in recent privacy-preserving encryption schemes of nonlinear dynamics and transform-domain processing, which strengthens the validity and reliability of the experimental methodologys.

The following example shows and describes the proposed cryptography process steps. Suppose the sending message is “This Message is Encrypted based on the Proposed Method.”

Plaintext:

This Message is Encrypted based on the Proposed Method

The first step: Character Encoding into DNA

GGTC CCTT GTTA GGCA GCCC ATGT TATC GGCA GGCA GGGC CCGG TATC GCCC GTTA GGCA GCCC TAAG CAAG TGAC TCCT CGGA GAAG GGTT TATC TTCT GCCC CTAT GGGC GGCA TATC TTCT GCCC CCGC CAAG GCCC GGTT CCTT TATC GCCC TGCA TCCT CCGC GAAG CCGC GGCA TATC TTCT GCCC ATGT TATC GGTT CCTT CCGC TTCT

The second step: Transcription to RNA

GGUC CCUU GUUA GGCA GCCC AUGU UAUC GGCA GGCA GGGC CCGG UAUC GCCC GUUA GGCA GCCC UAAG CAAG UGAC UCCU CGGA GAAG GGUU UAUC UUCU GCCC CUAU GGGC GGCA UAUC UUCU GCCC CCGC CAAG GCCC GGUU CCUU UAUC GCCC UGCA UCCU CCGC GAAG CCGC GGCA UAUC UUCU GCCC AUGU UAUC GGUU CCUU CCGC UUCU

The third step: RNA Complementary Base Pairing

CCAG GGAA CAAU CCGU CGGG UACA AUAG CCGU CCGU CCCG GGCC AUAG CGGG CAAU CCGU CGGG AUUC GUUC ACUG AGGA GCCU CUUC CCAA AUAG AAGA CGGG GAUA CCCG CCGU AUAG AAGA CGGG GGCG GUUC CGGG CCAA GGAA AUAG CGGG ACGU AGGA GGCG CUUC GGCG CCGU AUAG AAGA CGGG UACA AUAG CCAA GGAA GGCG AAGA

The fourth step: RNA Segment Scrambling

CAAU GGAA CCAG UACA CGGG CCGU CCGU CCGU AUAG AUAG GGCC CCCG CCGU CAAU CGGG GUUC AUUC CGGG GCCU AGGA ACUG AUAG CCAA CUUC GAUA CGGG AAGA AUAG CCGU CCCG GGCG CGGG AAGA CCAA CGGG GUUC CGGG AUAG GGAA GGCG AGGA ACGU CCGU GGCG CUUC CGGG AAGA AUAG CCAA AUAG UACA AAGA GGCG GGAA

The fifth step: Optional Reverse Transcription

CAAU GGAA CCAG TACA CGGG CCGU CCGU CCGU AUAG AUAG GGCC CCCG CCGU CAAU CGGG GUTC AUTC CGGG GCCU AGGA ACTG AUAG CCAA CUTC GATA CGGG AAGA AUAG CCGU CCCG GGCG CGGG AAGA CCAA CGGG GUTC CGGG AUAG GGAA GGCG AGGA ACGU CCGU GGCG CUTC CGGG AAGA AUAG CCAA AUAG TACA AAGA GGCG GGAA

The last step: Encoding into Character

nqhESZKnahSNfkSkqDqDhfSaSNnqFUkdqdaUvjDPtqDfEXdcKFUEqDSNSaUFaUmcSmFUkBaUJZhmUFZikSkhFjdaUEJDSmJDKCEUahE

Ciphertext:

nqhESZKnahSNfkSkqDqDhfSaSNnqFUkdqdaUvjDPtqDfEXdcKFUEqDSNSaUFaUmcSmFUkBaUJZhmUFZikSkhFjdaUEJDSmJDKCEUahE

According to the security point of view, the proposed bio-inspired cryptosystem offers a very large key space with random DNA encoding table (40 – possible mappings of long character sets) and RNA scrambling permutations. This renders brute-force attacks computationally infeasible. The proposed approach provides much uncertainty to attackers as compared to AES and RSA, which involve fixed mathematical structures, and additional biological randomness and the session-dependent encoding. Compared to AES, based on fixed block size, the offered approach encrypts text of varying size using dynamic encoding tables and does not allow the statistical analysis directly. Although RSA is highly secure in terms of asymmetry, it is computationally expensive to large messages. The suggested scheme proves to be competitive in terms of secure text communication, particularly in situations where there is need of lightweight and adaptive encryption.

## 4. Conclusion

The cryptography method inspired by bio-sequences introduced in this work is a secure and practical way to encrypt text based on DNA and RNA sequencing. The multi-step encryption method means that every message is encrypted in a very special manner that outsiders will not be able to decode. By evaluating the technique based on various message sizes, we determined that the method is fast and encryption and decryption times are reasonable even with large messages. The proposed system not only brings a novel view on cryptography but also opens up more possibilities for further studies on bio-inspired security solutions. This cryptographic technique is particularly useful in those functions that need to be highly secure and to be processed at a high speed, like, security in Internet communication and data transfer.
